# Rigid Polyurethane Foam Derived from Renewable Sources: Research Progress, Property Enhancement, and Future Prospects

**DOI:** 10.3390/molecules30030678

**Published:** 2025-02-04

**Authors:** Yao Yuan, Qinhe Guo, Lulu Xu, Wei Wang

**Affiliations:** 1 Fujian Provincial Key Laboratory of Functional Materials and Applications, School of Materials Science and Engineering, Xiamen University of Technology, Xiamen 361024, China; yuanyao@xmut.edu.cn (Y.Y.); blwdlx@163.com (Q.G.); 2 School of Chemical Engineering, University of New South Wales, Sydney, NSW 2052, Australia; 3 School of Mechanical and Manufacturing Engineering, University of New South Wales, Sydney, NSW 2052, Australia

**Keywords:** rigid polyurethane foam, environment friendly, bio-based polyols, modification, mechanical property, flame retardancy

## Abstract

Rigid polyurethane foam (RPUF) is a widely utilized thermosetting polymer across various industrial applications, valued for its exceptional properties. However, the demand for sustainable alternatives to petroleum-based polymers has grown increasingly urgent due to rising environmental concerns. Despite its widespread use, RPUF faces challenges such as inadequate mechanical strength, limited thermal stability, and high flammability, all of which are crucial considerations in commercial and household applications. Globally, ongoing efforts are focused on developing innovative technologies that convert renewable sources into new monomers and polymers, some of which could serve as alternatives to traditional RPUFs. Several approaches have been explored to improve the thermal stability, mechanical strength, and flame retardancy of RPUFs, including the modification of bio-based polyols and the incorporation of performance-enhancing fillers. This review emphasizes recent advances in RPUFs derived from natural resources, focusing on their preparation, characterization, and properties, and strategies to enhance the mechanical strength and flame safety of bio-based RPUFs. Additionally, it explores the applications of RPUF materials across various fields, addressing the challenges and potential developments in packaging, household items, construction, and automotive applications.

## 1. Introduction

Polymer foams, including polyurethane, polystyrene, polyvinyl chloride, and phenolic, are extensively utilized in various applications across insulation, packaging, construction, and the automotive industry [1,2]. Rigid polyurethane foam (RPUF) stands out among these materials for its low thermal conductivity, lightweight nature, excellent mechanical properties, and hydrophobic characteristics [3,4,5,6,7]. Recent data indicate that the global polyurethane market is expected to reach USD 88 million by 2026, with a compound annual growth rate (CAGR) of 6.0% [8]. Traditionally, RPUF is synthesized from polyols and isocyanates sourced from petroleum-based feedstocks. While these materials have facilitated the widespread adoption of polyurethane foams, their dependence on non-renewable fossil resources poses significant environmental and sustainability challenges. The production and disposal of petroleum-based polyurethanes contribute to resource depletion and environmental pollution.

Growing environmental concerns and the finite nature of fossil fuel resources have spurred significant efforts toward developing sustainable materials sourced from renewable resources [9,10]. In the last decade, significant progress has occurred in the development of bio-based RPUFs, primarily driven by advancements in green chemistry, polymer engineering, and materials science [11,12,13]. Researchers have investigated a diverse range of renewable feedstocks, such as vegetable oils, lignocellulosic biomass, sugar cane bagasse [14], corn starch [15], and wood [16], to synthesize polyols with properties specifically tailored for polyurethane applications [17]. Among these, vegetable oils, including soybean oil [18,19,20], castor oil [21,22,23], rapeseed oil [24,25,26], palm oil [27,28], sunflower oil [29,30], tung oil [31,32], and canola oil [33], have emerged as the most widely used renewable sources due to their abundance, making them ideal for developing bio-based polyurethane foam. Caillol et al. made significant strides in the development of fully bio-based polyurethane foams and their recycling [34]. The successful incorporation of these bio-based polyols into RPUF formulations has demonstrated that they do not easily achieve performance levels comparable to their petroleum-based counterparts. Consequently, significant efforts have been made to improve the overall properties of these RPUFs by incorporating various performance-enhancing fillers.

However, the transition from fossil fuel-based to bio-based polyurethane foams is not without its challenges. The inherent variability and complexity of natural feedstocks, coupled with the need for efficient and scalable production processes, have posed significant technical and economic hurdles. Additionally, the thermal, mechanical, and flame-retardant properties of bio-based RPUFs must be carefully optimized to meet the stringent requirements of industrial applications [35]. It is imperative that the mechanical properties are enhanced to provide the necessary durability and high mechanical strength for applications ranging from construction to automotive components. Additionally, flame-retardant properties are vital for safety compliance, requiring the incorporation of advanced anti-flaming agents and thorough testing to ensure that the foams resist ignition and slow fire spread [36,37]. The challenge lies in achieving a balance, where these properties are not only improved but also harmonized with the sustainable benefits of using bio-based materials.

To address these challenges, researchers have focused on property enhancement strategies, such as the chemical modification of bio-based polyols, incorporation of functional additives, and optimization of foam formulation and processing conditions. A key approach is the structural modification of bio-based polyols. Adjusting the chemical structure of these polyols allows researchers to precisely control the mechanical strength and flame safety of the foams. Another important strategy is incorporating functional components, including a range of fillers, stabilizers, and flame retardants, which can significantly enhance the performance of bio-based RPUFs. Additionally, optimizing foam formulation and processing conditions is crucial for achieving the desired properties in the final product. Through these comprehensive strategies, researchers aim to address the inherent challenges of bio-based RPUFs, facilitating their wider adoption across various industries and advancing the advancement of sustainable materials.

This paper intends to offer a comprehensive overview of the current research on RPUFs derived from renewable sources, with a specific focus on achieving the desired properties of RPUF. It will discuss the various types of bio-based polyols and isocyanates, along with the diverse fillers used in the production of RPUF, highlight the property enhancement strategies employed to improve foam performance, and explore the potential future prospects and challenges for the widespread adoption of bio-based RPUFs. This review aims to provide insights into the future potential of utilizing these fillers to enhance properties and provide timely updates on the progress toward developing sustainable materials for a greener future.

## 2. Approaches to Synthesis of Bio-Based RPUFs

With increasing environmental concerns and a growing demand for sustainable materials, bio-based RPUFs have attracted considerable attention in recent years. The synthesis of bio-based RPUFs primarily focuses on replacing traditional petroleum-based polyols and isocyanates with bio-based alternatives, such as vegetable oils, polysaccharides, lignins, and cellulose [38,39,40,41]. These bio-based polyols, often modified through chemical processes like esterification or epoxidation, improve the sustainability of the foam while maintaining the required performance properties [42,43,44]. Additionally, natural fillers like cellulose and lignin fibers are also incorporated to improve the mechanical strength, thermal insulation, and fire safety of RPUFs.

The synthesis of bio-based RPUFs often involves utilizing vegetable oils, which are abundant and renewable resources. However, most vegetable oils lack hydroxyl groups, a crucial functional group for polyol synthesis, making them unsuitable for direct use in foam preparation. Castor oil, however, is an exception, as it naturally contains hydroxyl groups (approximately 2.7 hydroxyl groups per triglyceride), making it a valuable polyol source for RPUF synthesis [38,45]. To overcome the limitations of other vegetable oils, functionalization methods such as epoxidation, hydroxylation, or transesterification are employed to introduce hydroxyl groups into the oils, thus enabling their use in polyurethane synthesis.

### 2.1. Transesterification

The transesterification approach is a widely used method for synthesizing bio-based RPUFs [46,47,48]. This process involves the reaction of vegetable oils, such as soybean, sunflower, or palm oil, with alcohols in the presence of a catalyst to produce alkyl esters and polyols. The polyols generated from transesterification have hydroxyl groups, which are essential for the formation of polyurethane linkages. By modifying factors like the type of alcohol, catalyst, and reaction duration, the molecular weight and functionality of the resulting polyols can be controlled, making them suitable for use in foam production. This method not only allows for the use of a wide range of renewable vegetable oils, but also helps in improving the properties of bio-based polyols, making the resulting RPUFs more sustainable, environmentally friendly, and adaptable for various applications. Silva et al. [49] used chemically modified tung oil as the main polyol component in the formulation of viscoelastic polyurethane foams. In this process, the tung oil was first hydroxylated through a two-step procedure. The initial step involved reacting tung oil with hydrogen peroxide solution and formic acid to produce hydroxylated tung oil (HTO). In the second step, this intermediate product was reacted with dry triethanolamine through a transesterification process, resulting in the formation of polyols.

### 2.2. Epoxidation

The epoxidation approach is another effective method for synthesizing bio-based RPUFs. This process involves the conversion of unsaturated vegetable oils into epoxy-functionalized oils, serving as polyol sources for RPUF synthesis. In the epoxidation process, the double bonds present in vegetable oils react with an oxidizing agent, resulting in the formation of an epoxide group. The resulting epoxidized oil contains reactive oxirane rings that can undergo further chemical modifications, such as hydroxylation or transesterification, to introduce hydroxyl groups or other functionalities suitable for polyurethane synthesis. Arniza et al. [50] developed a three-step process to synthesize transesterified palm olein-based polyols, involving transesterification with glycerol, epoxidation, and epoxide ring opening. The transesterification step introduced additional hydroxyl groups, enhancing the functionality of palm olein. Epoxidation converted the double bonds in the fatty acid chains into reactive epoxide groups, which were then opened to form a polyol suitable for polyurethane synthesis.

### 2.3. Other Approaches

The synthesis of bio-based polyurethane foams can be enhanced through various chemical modification approaches, including hydroformylation, amidation, and thiol–ene coupling [45,51,52]. Hydroformylation introduces formyl groups to unsaturated fatty acid derivatives, such as vegetable oils, by reacting them with carbon monoxide and hydrogen, creating aldehyde functionalities suitable for polyol synthesis. Amidation involves the reaction of polyols with organic acids or their derivatives, forming amide linkages that increase the reactivity and compatibility of bio-based polyols with isocyanates. Meanwhile, thiol–ene coupling utilizes the addition of thiol groups to alkenes in the presence of light or heat, forming thioether bonds that enhance the cross-linking and mechanical properties of the polyols. These modification techniques provide a sustainable way to create bio-based polyols with improved functionalities for the production of high-performance RPUFs with tailored properties for a variety of applications.

## 3. Preparation of Bio-Based RPUFs

The development of sustainable materials has been significantly advanced by RPUFs sourced from renewable resources. Bio-based RPUFs, produced from natural, renewable resources such as vegetable oils, lignocellulosic biomass, sugars, and other plant-derived materials, provide an eco-friendly option for producing RPUFs [42,53]. These renewable raw materials, particularly bio-based polyols from soybean oil, castor oil, palm oil, and other biomasses, are utilized to replace traditional petroleum-derived components in RPUF formulations. The transition towards bio-based RPUFs is in line with global efforts aimed at decreasing reliance on fossil fuels, promoting sustainable manufacturing practices.

### 3.1. Vegetable Oil-Based Polyurethane

Different types of vegetable oils with fewer hydroxyl groups are being explored for the development of environmentally friendly RPUFs. These foams are produced from oils such as soybeans, castor, palm, and other plant-based sources, which are chemically modified to produce polyols. However, the inherent hydroxyl group content in these vegetable oils is often insufficient to generate rigid polyurethane foam, affecting the reactivity of the foam and the extent of cross-linking within the polyurethane network [54]. To address this issue, it is essential to perform chemical modifications that increase the hydroxyl values of polyols derived from natural resources. Table 1 presents different techniques used to modify the fundamental structure of vegetable oils to raise their hydroxyl values, consequently enhancing the mechanical strength, thermal insulation properties, and rigidity of the resulting foam.

#### 3.1.1. Soybean Oil-Based Polyols

Soybean oil, an abundant and renewable resource, is recognized as an eco-friendly product with broad applications in the food and pharmaceutical industries. In RPUF applications, soybean oil plays a key role in developing bio-based polyols, which are essential for foam formulation. By chemically modifying soybean oil to increase its hydroxyl content, its effectiveness in the polymerization process required for creating rigid foams is significantly improved. The incorporation of soybean oil-derived polyols into RPUF formulations not only diminishes dependence on petroleum-based resources but also contributes to improved overall performance.

**Figure 1:**
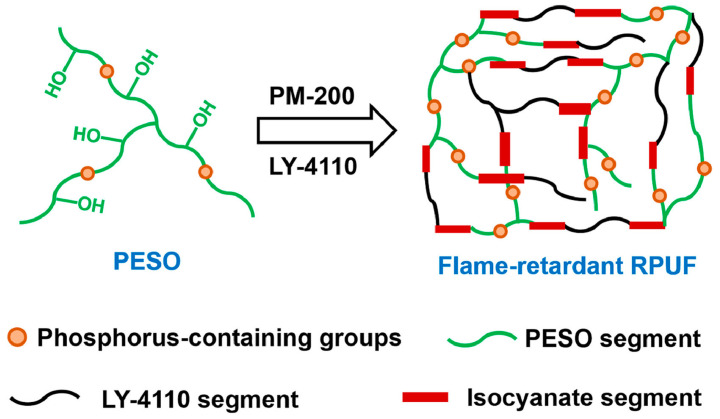
The preparation of the flame-retardant RPUF from PESO [62]. Copyright 2021. Reproduced with permission from Elsevier Science Ltd.

Petzhold et al. [61] synthesized a polyol from phosphorylated epoxidized soybean oil (ESO) with phosphorus contents of 2.55% and 1.32% by weight, demonstrating strong potential for use in flame-retardant RPUFs. The phosphorylated soybean oil-based polyols, Polyol-PN1 and Polyol-PN2, were utilized, with Polyol-PN1 replacing 75–80% of the traditional polyol, while Polyol-PN2 was used with a replacement range of 25–100%. Additionally, the study investigates the physical properties and fire safety of the foams produced with these bio-based polyols. As shown in Figure 1, Tang et al. [62] synthesized a bio-based flame-retardant polyol (PESO) through the phosphorylation of epoxidized soybean oil with trihydroxymethylphosphine oxide. The ESO-based polyols (PESO) were used to replace 10%, 20%, and 30% of the traditional flame-retardant polyol LY-4110. This replacement range was examined to explore how varying bio-polyol concentrations affect the characteristics of the resultant materials. This modification significantly reduced the flammability of RPUF, with the peak heat release rate and total smoke production of the flame-retardant RPUF containing 12.3 wt% PESO, decreasing by 40% and 49%, respectively.

#### 3.1.2. Rapeseed Oil-Based Polyols

Owing to its numerous advantages, such as high bio-safety, cost-effectiveness, biodegradability, and widespread availability, rapeseed oil-derived chemicals form an essential base for the development of bio-based polymers and additives. Zieleniewska et al. [24] incorporated a rapeseed oil-based polyol into RPUFs as a substitute for a petroleum-based polyol. The bio-based polyol was utilized to replace 25%, 50%, 75%, and 100% of the traditional polyol. The substitution led to the formation of RPUFs with reduced water absorption, enhanced thermal insulation, and improved microbiological resistance. However, the reduced cross-linking network of the bio-based RPUFs resulted in decreased mechanical properties. Uram et al. [63] developed bio-polyols from rapeseed oil through epoxidation and subsequent ring opening with 1-hexanol and 1,6-hexanediol, and applied them to rigid polyurethane foams. The findings showed that increasing the bio-polyol content led to a reduction in both the apparent density and mechanical properties of the RPUFs. Kairyte et al. [64] developed rapeseed oil-based polyols modified with propylene glycol and glycerin. They discovered that the use of a glycerol-modified bio-polyol produced RPUFs with enhanced cross-linking density and improved thermal insulation properties.

#### 3.1.3. Castor Oil-Based Polyols

**Figure 2:**
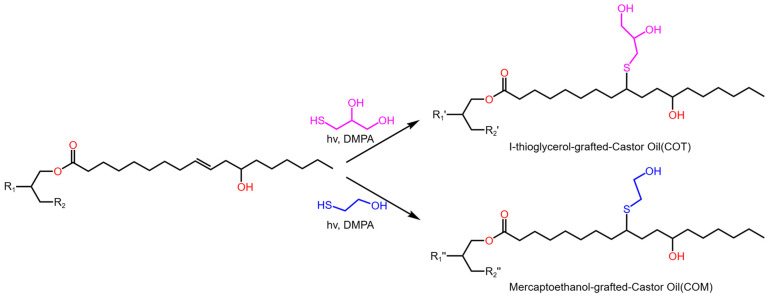
Preparation of castor oil-based multifunctional polyols using the thiol–ene photo-click reaction.

Castor oil is highly biodegradable and possesses a high hydroxyl functionality, which makes it an excellent option for synthesizing polyols. Its low toxicity and wide availability further contribute to its suitability for use in RPUF formulations. These castor oil-derived polyols provide unique properties that enhance the overall performance of the resulting RPUFs. As illustrated in Figure 2, Lee et al. [65] synthesized castor oil (CO)-based polyols with high functionality using a facile thiol–ene click reaction, followed by the preparation of polyurethane foams via a foaming process. The CO-based polyol replaced commercial polyols at different ratios of 25%, 50%, 75%, and 100%. The resulting foams exhibited substantial improvement in mechanical properties, with compressive strength increased by 75% and a marked enhancement in thermal stability. Wang et al. [38] developed two bio-based RPUFs incorporating modified polyols derived from castor oil, resulting in significant enhancements in both insulation efficiency and flame retardancy. The BIO2 composite achieved a limiting oxygen index (LOI) of 27.2 vol% and a V-0 rating in the vertical burning test. Additionally, the compressive strength of the BIO2 foam improved by 57% compared to BIO1, a benefit stemming from the effective interfacial adhesion between the graphene oxide structure and the RPUF matrix.

#### 3.1.4. Tung Oil-Based Polyols

**Figure 3:**
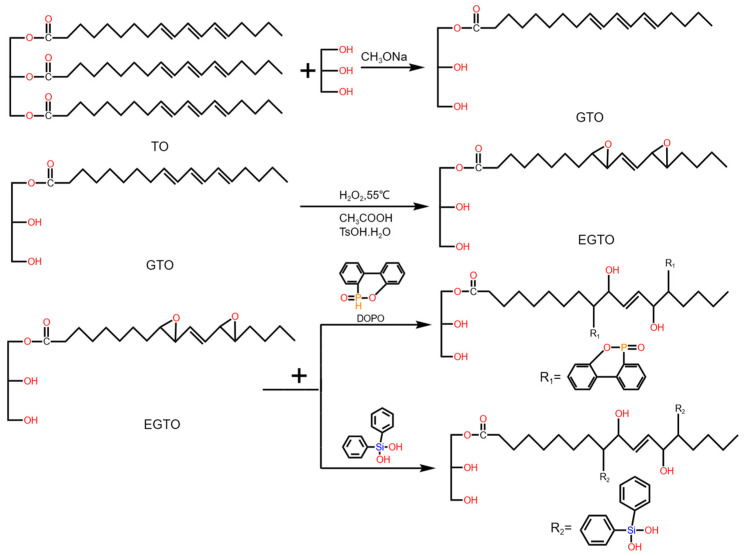
Synthesis route of tung oil monoglyceride (GTO), epoxidized monoglyceride of tung oil (EGTO), phosphorus-containing tung oil-based polyol (PTOP), and silicon-containing tung oil-based polyol (PTOSi).

Tung oil, a natural drying oil characterized by its high degree of unsaturation, is recognized as a precious bio-based material suitable for a range of applications, including the production of bio-polyols used in RPUFs [66,67]. As portrayed in Figure 3, Zhang et al. [67] developed phosphorus- and silicon-containing tung-based polyols for producing RPUFs through a three-step process: (i) transesterification of tung oil, (ii) epoxidation, and (iii) epoxide ring opening with 9,10-dihydro-9-oxa-10-phosphaphenanthrene (DOPO) and dihydroxydiphenylsilane (DPSD), respectively. The resulting polyols (PTOP and PTOSi) were used to produce RPUFs and were shown to be effective flame retardants. The LOI values for RPUFs with 80% PTOP and PTOSi reached 24.0% and 23.4%, respectively. Interestingly, a higher polyol content led to increased thermal conductivity, but reduced foam density.

### 3.2. Polysaccharide-Based Polyurethane

The preparation of bio-based RPUFs from polysaccharides involves utilizing natural, renewable polysaccharide sources like starch, cellulose, and chitosan to produce bio-polyols, garnering increasing attention and research within the polyurethane matrix. The process typically involves chemical modification of the polysaccharides to generate polyols with the required hydroxyl functional groups needed for polyurethane formation. Polysaccharides hold significant potential in the development of bio-based RPUFs, with promising applications across various industries driven by the growing focus on sustainability and performance enhancements.

#### 3.2.1. Starch-Based Polyurethane

Starch-based RPUFs represent a novel category of bio-based materials that utilize starch, a renewable polysaccharide, as a primary feedstock for polyol production. Starch is chemically modified, typically through hydrolysis, esterification, or etherification, to introduce hydroxyl groups that react with isocyanates, forming the polyurethane matrix. These starch-derived RPUFs offer a sustainable alternative that contrasts conventional petroleum-based RPUFs, with the added benefits of being biodegradable and derived from abundant, renewable resources. Recent advancements in starch modification and foam formulation are enhancing the overall properties of starch-based RPUFs, expanding their potential use across various industrial applications.

Lubcazk et al. [68] synthesized polyetherols from starch by reacting it with allyl carbonate, and used these polyetherols to produce RPUFs. The resulting foams exhibited similar apparent density, water uptake, and polymerization shrinkage compared to conventional RPUFs, while demonstrating enhanced thermal resistance, being capable of withstanding prolonged exposure to temperatures of up to 175 °C. Additionally, thermal exposure improved the compression strength of these RPUFs, highlighting their promising potential for use in insulation materials. Lubcazk et al. [69] utilized starch as a raw material, along with formaldehyde, glycerol, and allyl carbonate as functionalizing agents, to synthesize oligomers for use in RPUF production. The resulting polyurethane foams exhibited excellent heat resistance, with enhanced mechanical properties after thermal exposure and the ability to withstand prolonged heating at temperatures of up to 200 °C.

#### 3.2.2. Chitosan-Based Polyurethane

Chitosan-based RPUFs are an innovative class of bio-based materials derived from chitosan and obtained through the deacetylation of chitin [70,71]. Chitosan consists of N-acetylglucosamine and glucosamine units connected by β-1,4-glycosidic bonds. Its rich content of hydroxyl and amino groups makes it highly suitable for the preparation of bio-based polyols. These polyols are essential in the synthesis of RPUFs, imparting unique properties such as enhanced biodegradability, improved mechanical strength, antimicrobial activity, and thermal stability.

Strzalka et al. [72] prepared hydroxylated chitosan by using glycerol and ethylene carbonate under various conditions to produce RPUF. The foam exhibited performance comparable to conventional RPUFs, with improved thermal resistance and increased compressive strength following heat treatment at 150 °C. Javaid et al. [73] synthesized starch/chitosan-modified polyurethane using an -NCO end-terminated prepolymer and a chain extender composed of 1,4-butanediol, starch, and chitosan. They found that when the amounts of starch and chitosan were balanced, the polyurethane exhibited enhanced thermal stability. This balanced combination of starch and chitosan proved to be an effective approach for preparing the polyurethane.

#### 3.2.3. Cellulose-Based Polyurethane

Cellulose, originating from natural materials like wood and plant fibers, is chemically modified to create bio-based polyols that are crucial for the synthesis of RPUFs. Utilizing cellulose in RPUFs contributes notably to the development of eco-friendly, high-performance materials. Maiuolo et al. [74] formulated innovative bio-based polyurethane composite foams by using cellulose-derived polyols as chain extenders and citric acid cellulose as a thickener. The hyperbranched structure of citric acid cellulose enhanced hydrogen bonding within the polyurethane matrix, leading to a denser and more compact foam structure. The resulting bio-based polyurethane foams and their composites exhibited strong mechanical properties. Szpilyk et al. [75] synthesized polyols using cellulose, triglycerides, and ethylene carbonate in water and then used these polyols to make rigid polyurethane foam. The resulting foam showed excellent density, water absorption, polymerization shrinkage, and heat resistance. Additionally, the biodegradable nature of the polyols used resulted in a polyurethane foam with inherent biodegradability.

### 3.3. Lignin-Based Polyurethane

**Figure 4:**
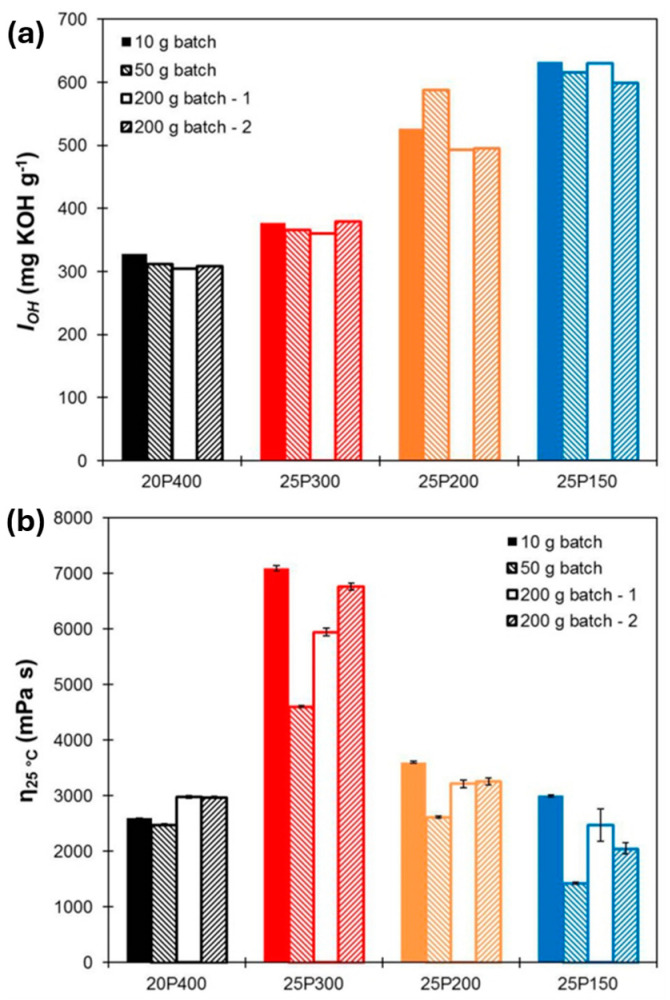
Hydroxyl value I_OH_ (**a**) and viscosity η at 25 °C (**b**) of the polyols prepared on different scales [81].

Lignins are complex organic polymers that, alongside cellulose and hemicellulose, form the structural framework of the plant cell wall. As the second most abundant renewable source of aromatic carbon, lignins have a highly complicated structure [76]. Their reactivity can be enhanced through chemical modifications, such as graft polymerization and epoxidation [77,78,79], enabling them to partially substitute petroleum-based materials [80]. Duval et al. [81] synthesized a highly reactive lignin-based liquid polyol by reacting organosolv lignin with ethylene carbonate. Figure 4 presents a comparison of the I_OH_ and viscosities of the polyols prepared on various scales. The results indicate that polyurethane foams made with a 25 wt.% replacement of petroleum-based polyols exhibited properties comparable to those of commercial foams. In a separate study by Hatakeyama et al. [82], RPUFs were made from lignin/molasses-based polyols at varying NCO/OH ratios. The results indicated that, depending on the lignin-to-molasses ratio, these RPUFs could serve as effective thermal insulation materials, with thermal conductivity values ranging from 0.030 to 0.040 W·m^−1^·K^−1^.

**Figure 5:**
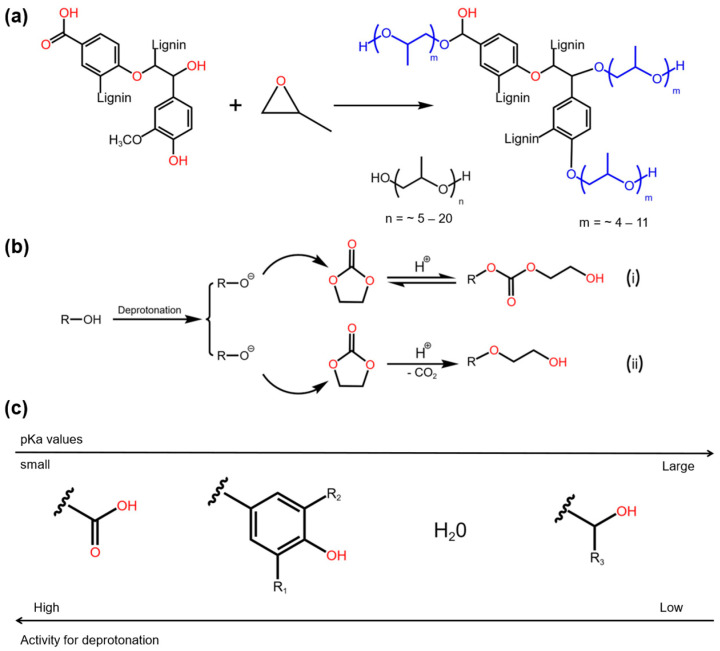
(**a**) Oxyalkylation of an industrially isolated lignin with propylene oxide for bio-polyol production, (**b**) mechanism of ethylene carbonate ring opening reactions, and (**c**) deprotonation of different hydroxyl groups.

Lignocellulosic materials, such as lignin derivatives, straws, barks, bagasse, and wood, are frequently used in the preparation of bio-based RPUFs [40,83]. Zhang et al. [84] found that replacing approximately 25 wt.% of conventional petroleum-based polyols with polyols derived from liquefied rice straw, wheat straw, oilseed rape straw, or corn straw resulted in RPUFs exhibiting improved thermal conductivity and comparable mechanical properties. Similarly, as shown in Figure 5, Xuefeng et al. [85] found that using oxyalkylated kraft lignin in RPUFs improved the mechanical performance when bio-polyols were used instead of petroleum-based polyols. Li et al. [86] synthesized RPUFs from fractionated lignin with high functionality and low molar mass, using ethanol-organic fractionation of corn straw to isolate the lignin. Lignin precipitated at 30% or 15% ethanol showed a lower molecular weight and higher active hydroxyl content, resulting in foams with enhanced compressive strength (0.78 ± 0.02 MPa), reduced thermal conductivity (0.037 ± 0.01 W·m^−1^·K^−1^), and improved thermal stability.

## 4. Natural Filler Reinforcement in RPUFs

A promising strategy for making RPUFs more environmentally friendly is reinforcing polymeric materials with natural fillers sourced from forestry or agricultural waste, aligning with circular economy principles. These bio-based fillers, including materials like wood fibers, straw, husks, and other plant residues, contribute to reducing dependence on petroleum-based components. By incorporating natural fillers, the mechanical properties of RPUFs, such as stiffness and durability, can be improved, and the reactive groups in these fillers can also interact with isocyanates. Various fillers derived from agricultural and forestry waste have been explored.

### 4.1. Cellulose Filler Reinforcement in RPUFs

**Figure 6:**
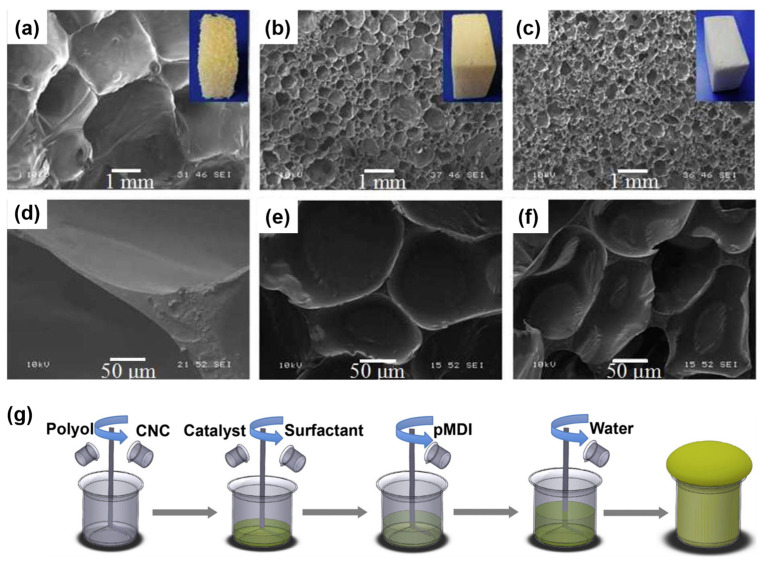
(**a**–**f**) cell morphology and appearance of prepared foams (**a**) PPU, (**b**) BPU, and (**c**) BPU4 nanocomposite and detailed views showing cell wall and strut area of (**d**) PPU, (**e**) BPU and (**f**) BPU4 nanocomposite. (**g**) Schematic diagram of the lab-scale foaming process of BPU foams and nanocomposite foams [27]. Copyright 2016. Reproduced with permission from Elsevier Science Ltd.

Cellulose fillers, including nanocrystals, fibers, and microcellulose, have attracted considerable interest as reinforcement materials in RPUFs. Zhou et al. [27] synthesized RPUFs by incorporating cellulose nanocrystals into a palm-oil polyol. The RPUFs were fabricated following the process outlined in Figure 6g, and the microstructures of petroleum polyurethane (PPU) foams, water-blown biopolyurethane (BPU) foams, and BPU4 (containing 4 phr of cellulose nanocrystals) foams are shown in Figure 6a–f. The resulting RPUF composites demonstrated enhanced compressive strength and superior dimensional stability under both freezing and heating conditions compared to unmodified foams. With an increase in cellulose content, the rigidity of the RPUFs correspondingly rose, which led to reduced deformation resilience. Uram et al. [87] investigated the reinforcement of RPUFs with 1, 2, and 3 wt.% of cellulose filler. The addition of this natural filler influenced the cellular structure of the composites, resulting in a uniform structure and a high content of closed cells (>90%). Kurańska et al. [88] investigated the effect of incorporating varying amounts of microcellulose (3, 6, and 9 wt.%) on the physicomechanical properties of RPUFs made from a rapeseed oil-based polyol. The addition of this natural filler led to a significant increase in the flammability of the modified foams, attributed to the inherently high flammability of microcellulose. However, the addition of microcellulose at various levels had no effect on the apparent density of the RPUFs and positively impacted their mechanical performance.

### 4.2. Lignin Filler Reinforcement in RPUFs

**Figure 7:**
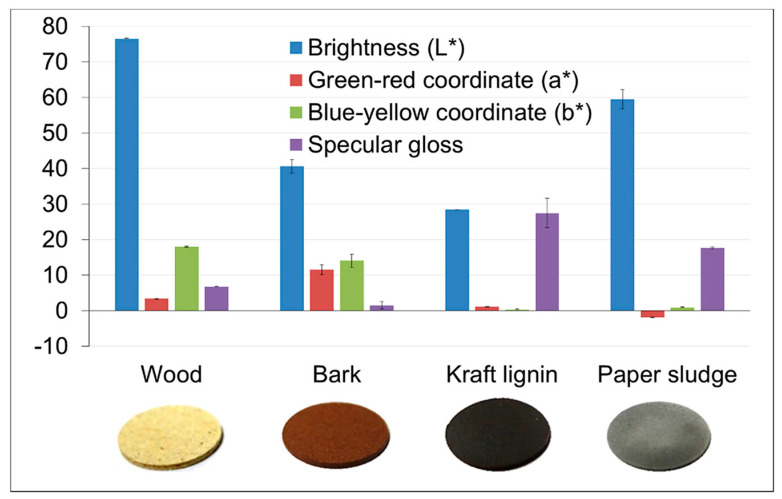
Optical properties and aspect of the four forest-based fillers [89]. Copyright 2019. Reproduced with permission from Elsevier Science Ltd.

Lignin fillers have been explored as reinforcement materials in RPUFs to enhance their properties and reduce reliance on petroleum-based polyols. As seen in Figure 7, Delcius et al. [89] presented noteworthy findings on RPUFs reinforced with various forestry wastes, including bark, wood, kraft lignin, and paper sludge. The visual and optical properties of various forest-based fillers in pellet form are shown. The wood filler is the brightest and most yellowish, due to non-structural compounds like polyphenols and flavonoids. Kraft lignin appears dark and colorless, with low L* and a* values. The bark pellet is brown with reddish hues, likely due to its high lignin content. Paper sludge has a grayish color, with low a*, due to oxides like calcium and aluminum. The specular gloss is highest for kraft lignin, followed by paper sludge, linked to the phenolic groups in lignin and oxides in paper sludge. The study revealed that adding pine bark is advantageous for RPUFs that demand high ultraviolet resistance. In contrast, the incorporation of wood chips resulted in RPUFs having a lower apparent density and enhanced photodegradation effects compared to the darker samples. Anwar et al. [90] found that incorporating pulp into RPUFs results in reduced water absorption. Specifically, RPUFs modified with 4 wt.% pulp exhibited satisfactory water absorption (11.5%) and water retention (75.9%) properties, which are linked to the cell structure of foams.

### 4.3. Other Industrial By-Product Reinforcement in RPUFs

RPUFs reinforced with 5–35 wt.% of the grain fraction of fly ash were developed by Paciorek-Sadowska et al. [91]. Compared to unmodified foams, the resulting composites showed reduced brittleness and increased apparent density, which enhanced their mechanical performance. In another work by Kairyte et al. [92], RPUFs were reinforced with 10–50 wt.% bottom waste ash. The ash appeared to act as a plasticizer, increasing viscosity and decreasing reactivity, as indicated by a lower foaming temperature. Foams with up to 10 wt.% filler had a uniform structure with consistent cell shapes. However, higher amounts resulted in irregular structures with visible loose particles. While adding 10–40 wt.% filler enhanced the thermal and mechanical properties relative to unfilled foams, 50 wt.% filler led to a deterioration in these properties.

Recently, the incorporation of conventional and fluidized fly ashes as fillers in polyurethane production has gained attention due to its potential to improve the overall properties of the material while promoting sustainability [93,94,95]. Fluidized fly ash, produced under controlled conditions, offers a more uniform particle size and improved dispersibility in polymer matrices, contributing to better performance in polyurethane products. Kuźnia et al. [96] modified PUR foam with up to 20 wt.% fluidized bed combustion fly ash, improving thermal stability and reducing carbon content and calorific values. Additionally, the incorporation of microspheres, which are hollow spherical particles, presents another promising approach. Microspheres, often made from materials such as glass, ceramic, or polymers, can be used to reduce the density of polyurethanes, leading to lightweight foams with excellent thermal insulation properties [97,98,99,100,101]. Furthermore, the integration of biomass-derived fly ash, which is a byproduct of biomass combustion, has been explored as an alternative to conventional fly ash [102,103]. These bio-based ashes offer a more sustainable solution, meeting the increasing need for eco-friendly materials. Anna Magiera et al. [104] explored the application of biomass-derived fillers, specifically soybean husk ash, in the production of RPUFs to reduce reliance on expensive chemicals. The study suggests that the biomass-derived ash had minimal impact on the cellular or chemical structure of the polyurethane matrix, indicating its potential for use as a sustainable filler in RPUF production without compromising key properties.

## 5. Application of Bio-Based RPUFs

Bio-based rigid polyurethane foams present promising potential for various uses because of their sustainability and environmental advantages. Sourced from renewable biomass, they provide an effective substitute for traditional petroleum-based polyurethanes, helping to meet the increasing demand for reducing dependence on fossil fuels. Bio-based polyurethanes offer several key advantages, including biodegradability, chemical resistance, and improved mechanical properties [105,106,107]. Their versatility is further enhanced by the incorporation of bio-based polyols and natural fillers, broadening their potential across various industries.

**Figure 8:**
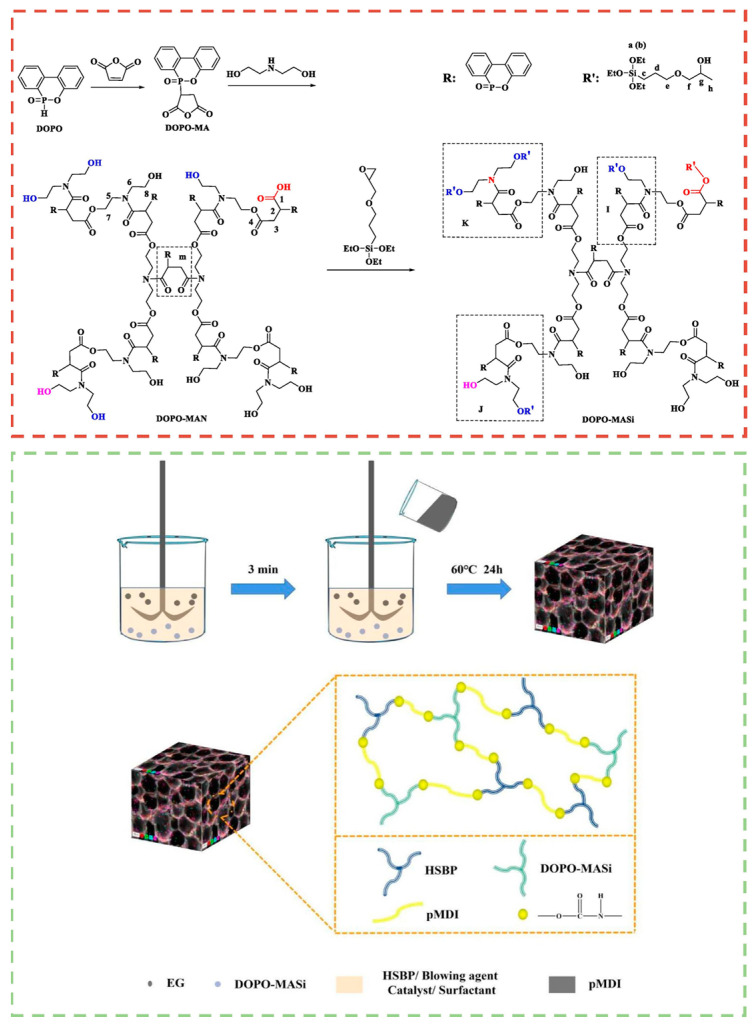
The preparation route and structure of flame-retardant RPUFs [110]. Copyright 2023. Reproduced with permission from Elsevier Science Ltd.

RPUFs are produced by incorporating flame retardants such as halogen-free, phosphorus-based, or nitrogen-based compounds into the bio-based RPUF matrix. These flame-retardant additives enhance the thermal stability and fire resistance of the material, making it suitable for applications where fire safety is critical [108,109]. As presented in Figure 8, Gong et al. [110] developed a reactive hyperbranched flame-retardant polyol incorporating three flame-retardant elements: phosphorus (P), nitrogen (N), and silicon (Si). This polyol was combined with expandable graphite (EG) to produce a plant oil-based RPUF featuring multiple flame-retardant systems. The resulting foam demonstrated outstanding flame-retardant and smoke-suppression capabilities, achieving a LOI of 30%. Ding et al. [111] developed a novel bio-based polyol from castor oil that includes both phosphorus and nitrogen. The peak heat release rate (pHRR) decreased by 51.5% compared to the pristine RPUF in cone calorimetry tests. This improvement in flame retardancy is due to the synergistic effect between phosphorus and nitrogen, which facilitated the formation of substantial residual chars.

In the construction industry, bio-based RPUFs are extensively utilized, particularly for insulation and thermal barrier applications. Their superior thermal insulation properties enable substantial reductions in energy consumption within buildings, thereby improving energy efficiency and adhering to green building standards [112]. Additionally, these foam materials exhibit good fire resistance, contributing to the overall safety of the structures. Akdogan et al. [113] developed bio-based RPUFs using a novel active phosphorus sunflower oil-based bio-polyol, incorporating expandable graphite (EG) and Dimethyl methyl phosphonate (DMMP) to enhance their flame retardancy. The thermal conductivity coefficients of the bio-based RPUF composites ranged from 28.72 to 32.51 mW/(mK), and the LOI value achieved 25.7%, which is approximately 32% higher than that of a petroleum-based RPUF for comparison. Zemla et al. [114] utilized rapeseed oil as a raw material to produce RPUFs incorporating bio-based polyols along with various phosphorus-containing flame retardants, such as triethyl phosphate (TEP), dimethyl phosphate propanoate (DMPP), and cyclic phosphoric acid esters. The resulting foam materials exhibited low thermal conductivity coefficients and limiting oxygen indexes (LOIs) exceeding 21 vol%. The modified RPUFs exhibited a reduced tendency toward fire development, classifying them as thermal insulation materials suitable for exterior wall applications, as well as self-extinguishing materials.

Bio-based RPUFs offer significant application potential in various areas, including sound absorption and electromagnetic shielding materials. As electronic devices proliferate, the need for materials that can mitigate electromagnetic interference (EMI) becomes increasingly critical. Bio-based RPUFs can be engineered to create effective barriers against EMI, safeguarding sensitive electronic equipment and ensuring optimal performance. Selvaraj et al. [115] developed a bio-based, low-density electromagnetic interference (EMI) shielding material using castor oil-based polyurethane, graphite nanosheets (GNPs), zirconia (ZrO_2_), and bamboo charcoal (BC). This material achieved a maximum EMI shielding effectiveness (SE) of 28.03 dB within the 8–12 GHz frequency range.

## 6. Concluding Remarks and Future Aspects

Bio-based rigid polyurethane foams (RPUFs) represent a promising alternative to traditional petroleum-derived materials, addressing the growing demand for sustainable solutions in various industries [116]. This review has underscored the significant advancements in the development and modification of bio-based RPUFs, highlighting their preparation methods and characterization and the incorporation of bio-based polyols and performance-enhancing fillers to enhance their overall properties. Additionally, it discusses the existing use of bio-based RPUFs across different areas and examines their future advancements and potential applications. Despite the progress made, challenges such as low mechanical strength and high flammability still need to be addressed to ensure the widespread adoption of these materials.

One major challenge is the variability in the properties of renewable materials, which may result in inconsistencies in the performance of the final products. For bio-polyols, this variability can lead to deteriorations in essential characteristics like mechanical strength and fire safety, which may fall short compared to petroleum-based counterparts, potentially limiting their use in demanding applications. Additionally, the compatibility of natural fillers like cellulose and lignin with polyurethane matrices is another hurdle, as achieving optimal dispersion and adhesion can be difficult. Recently, non-isocyanate-based polyurethane (PU) foam has garnered significant attention due to its eco-friendly characteristics, positioning it as a potential next-generation technological approach for producing high-performance and environmentally sustainable PU foam [117]. Another challenge lies in the availability and cost of sustainable feedstocks. Although there is increasing interest in using renewable resources, the current supply chain for these materials may not be fully established, leading to higher production costs. Furthermore, the compatibility of natural fillers, such as cellulose and lignin, with polyurethane matrices can pose difficulties in achieving optimal dispersion and adhesion, impacting the overall performance of the foams.

University studies have a critical role to play in tackling the environmental and industrial challenges associated with bio-based RPUFs. Research efforts should focus on developing scalable, cost-effective technologies that can improve the mechanical, thermal, and flame-retardant properties of bio-based foams. Additionally, universities can drive innovation by exploring new feedstock sources and sustainable production techniques, ensuring the future viability of bio-based RPUFs. These advancements will be crucial for bridging the gap between laboratory-scale research and industrial application. From an industrial perspective, the widespread commercialization of bio-based RPUFs depends on overcoming challenges related to raw material availability, cost, and performance. To this end, universities must collaborate with industry partners to facilitate the transition from research to real-world applications. Furthermore, continued efforts in developing environmentally friendly and sustainable processes will ensure that bio-based RPUFs not only meet industrial performance standards, but also contribute to broader sustainability goals.

In conclusion, to advance the development of bio-based RPUFs, several strategic efforts should be prioritized. First, future efforts for bio-based RPUFs should focus on optimizing their inherent biocompatibility by using advanced molecular design and modification techniques. Second, it is crucial to expand the supply chain for sustainable feedstocks, which involves investing in agricultural practices that support the growth of bio-based materials and exploring alternative biomass sources that can be sustainably harvested. Third, conducting comprehensive life cycle assessments will offer important information about the environmental impact of bio-based RPUFs in comparison to traditional materials, thereby supporting the formulation of effective marketing strategies and ensuring regulatory compliance [11]. By concentrating on these areas, future efforts can significantly enhance the functionality and applicability of bio-based polyurethanes, establishing a solid foundation for ongoing research and innovation in fields such as construction and insulation.

## Figures and Tables

**Table 1:** Modification methods for the development of polyols from vegetable oils.

Matrix	Bio-Based	Modification Method	Ref.
Thermoplastic polyurethane	Soybean oil-based polyols	Thiol–ene coupling	[55]
Polyurethane film	Soybean oil-based polyols	Epoxidation followed by oxirane ring opening	[56]
Rigid polyurethane foam	Castor oil-based polyols	Alcoholysis with triethanolamine	[57]
Rigid polyurethane foam	Castor oil-based polyols	Transesterified using triethanolamine	[58]
Rigid polyurethane foam	Palm olein-based polyols	Tranesterification followed by epoxidation and ring opening	[50]
Rigid polyurethane foam	Soy-based polyols	Oxirane ring opening via alcoholysis using methanol and ethylene glycol	[59]
Polyurethane coating	Sunflower oil-based polyols	Thiol–yne coupling	[60]

## Data Availability

Not applicable.
